# A Rectourethral Fistula due to Transrectal High-Intensity Focused Ultrasound Treatment: Diagnosis and Management

**DOI:** 10.1155/2012/962090

**Published:** 2012-12-09

**Authors:** Valeria Fiaschetti, Guglielmo Manenti, Isabelle Di Poce, Maria Fornari, Aurora Ricci, Enrico Finazzi Agrò, Giovanni Simonetti

**Affiliations:** ^1^Department of Diagnostic Imaging, Molecular Imaging, Interventional Radiology and Radiation Therapy, University Hospital Tor Vergata, Viale Oxford 81, 00133 Rome, Italy; ^2^Department of Urology, University Hospital Tor Vergata, Viale Oxford 81, 00133 Rome, Italy

## Abstract

Colovesical fistula (CVF) is an abnormal connection between the enteric and the urinary systems. The rectourethral fistula (RUF) is a possible but extremely rare complication of treatment of prostate cancer with “transrectal High-Intensity Focused Ultrasound (HIFU) treatment.” We present a case of CVF due to HIFU treatment of recurrent prostate cancer. The case was assessed with cystography completed with a pelvic CT scan—with MPR, MIP, and VR reconstruction—before emptying the bladder. Since the CT scan confirmed that the fistula involved solely the urethra and excluded even a minimal involvement of the bladder, it was possible to employ a conservative treatment by positioning a Foley catheter of monthly duration, in order to allow the urethra to rest. Still today, after 6 months, the patient is in a good clinical condition and has not shown yet signs of a recurrence of the fistula.

## 1. Introduction

Colovesical fistula (CVF) is an abnormal connection between the enteric and the urinary systems. Some authors believe that the first description of a colovesical fistula (CVF) was by Rufus of Ephesus in AD 200 [1]. Others say that the first complete description of CVF was thought to be given by Jones in 1858; but Harrison Cripps published the first monograph on 63 cases in 1888 [2].

CVF is the most frequent enterovesical fistula and usually occurs in the sigma, followed by rectum. It is a relatively rare but challenging complication of inflammatory diseases, such as diverticulitis and Crohn's disease, and of neoplastic conditions such as colorectal carcinoma and bladder carcinoma. CVF may also be a resulting complication of iatrogenic conditions from surgical procedures, such as prostatectomy and laparoscopy prostatectomy, from irradiation, and from High-Intensisty Focused Ultrasound (HIFU). CVF may also follow some traumatic conditions [1, 3]. According to some studies, diverticulitis is, at the moment, the most frequent cause of CVF (50–79%) [4–7]; others focus more on neoplastic pathologies. One particular study found that 38/41 CVF cases were associated with malignancy (92.7%), of which 15 (39.5%) were found to have tumor recurrence [3, 8].

The diagnosis is essentially clinical [4]. Typical symptoms are fecaluria (43–65%), pneumaturia (67–85%), and urine leakage from the rectum during voiding (40%). Fecaluria is a pathognomonic sign and is not usually present until late in the course of the disease when the fistula has become quite large [9]. Pneumaturia may not always be present in CVFs [9]. It may have other causes such as iatrogenic instrumentation or gasproducing organisms in the urinary tract, especially in diabetics [9, 10]. Other symptoms may be recurrent nonspecific urinary tract infection (UTI) 73%, abdominal pain (22%), and dysuria (14.6%) [3, 4, 8, 9]. 

Early diagnosis of enterovesical fistula is difficult [3]. It is very important to not only diagnose the fistula, but also to localize it correctly in the event of surgery [1]. In every occurrence but especially in the case of a suspected neoplastic pathology, it is necessary to establish the cause of the fistula to design and plan the best therapeutic and/or surgical approach of the treatment [11]. It is therefore necessary to use the available diagnostic technologies to confirm the diagnosis and prove the fistulous tract. 

We present a case of CVF due to HIFU treatment of recurrent prostate cancer. The case was assessed with cystography completed with a pelvic CT scan before emptying the bladder, in order to exclude even a minimal involvement of the bladder. In this way, it was possible to effect a conservative treatment of the fistula, with excellent results.

## 2. Case Presentation

A seventy-year-old patient underwent a prostatectomy due to the presence of a prostate cancer (cT3) in 2005, at the urology unit of our hospital and was discharged in good clinical condition. He underwent varians cycles of radiotherapy (RT). 

Given that, during the periodical followup, an increase in the values of PSA was detected, it was decided to submit the patient to a pelvic CT scan, in which a node was found, near the anastomosis. For this reason, the patient was once more checked in to the urology unit of the same hospital, in order to undergo HIFU treatment with Ablatherm (EDAP-TMS) in a single, one-hour session. 

During the first post-HIFU checkups, the patient complained of burning upon urination and mictional urgency with a reduced flow of urine, as well as outflow of urine from the anus. For this reason, he was once more checkedin to the hospital, to undergo radiological exams, under the suspicion that he had a urethrorectal fistula (RUF). A catheter was placed in his bladder. 

**Figure 1:**
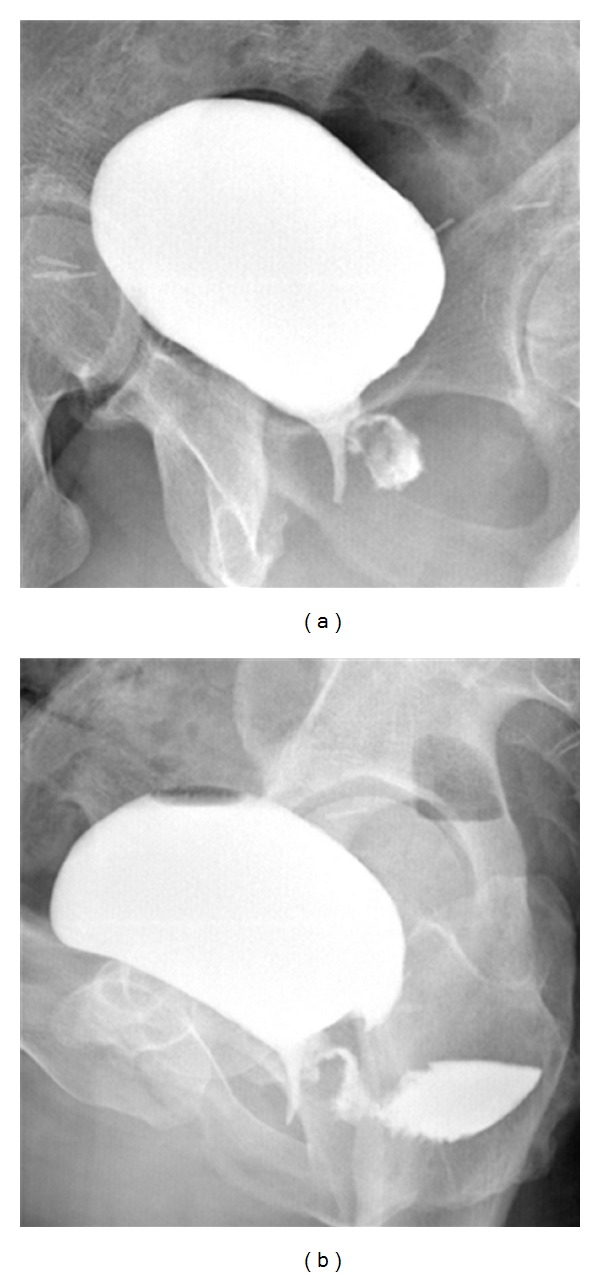
Retrograde cysto-uretrography. Oblique radiograms of bladder after full repletion with contrast media (a and b). The expansion of contrast media could be better observed in the oblique projections (a), especially when acquired after the space of a few minutes (b). In delayed oblique projection, as shown fistulous tract was more visible, as well as a certain amount of contrast in the rectum. It was not, however, possible to exclude with absolute certainty a partial involvement of the bladder.

Since the cystography is one of the most sensitive diagnostic exams for confirming the suspicion of a fistula, and the patient had already been catheterized, it was decided to carry out, as the initial radiological exam, a retrograde cystourethrography (Figure 1), which allowed us to observe an “expansion of the media contrast at the neck of the bladder.” Before emptying the bladder of the contrast media, it also was decided to supplement this exam with a CT scan of the pelvis. This exam was done in order to be sure that the bladder was not involved in the problem and that only the urethra was affected by the fistula. This was necessary in order to carry out a conservative treatment.

### 2.1. Cystouretrography

After having obtained an antro-posterior (AP) radiogram in basal condition, we proceeded to gradually fill the bladder with 150 cc of organoiodate contrast media (Omnipaque 350 mg I/mL, GE healthcare) and 150 cc of physiological solution. When the bladder was completely full, we effected AP and oblique radiograms (Figure 1). 

### 2.2. Pelvic CT with Three-Dimensional (3D) Reconstruction

**Figure 2:**
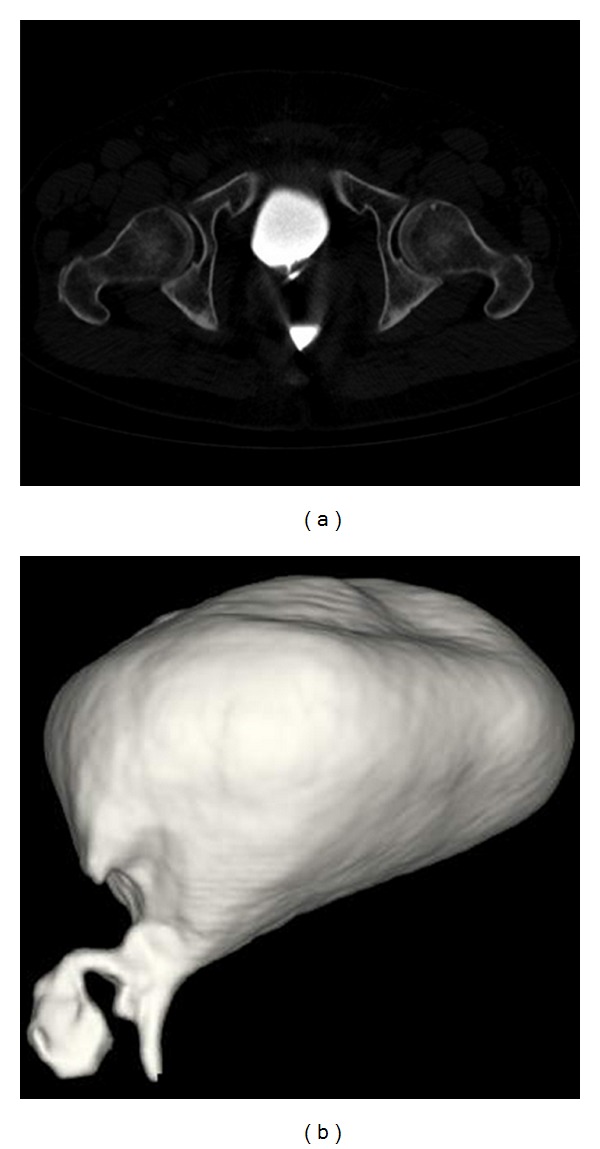
Pelvic CT scan with VR reconstruction. Pelvic CT scan was acquired before emptying the bladder of the contrast media, in order to confirm and to be sure that the bladder was not involved in the problem and that only the urethra was affected by the fistula.(a)Axial plane. (b)VR reconstruction.The latterallows to see very well the fistolous tract that connects the bladder to the rectum.

the CT scan of the pelvis was done using a 64-slice MDCT scanner (LightSpeed VCT, General Electric Medical System, Milwaukee, WI, USA) without administering i.v. and oral contrast media. All the images were acquired with the patient in a supine position and holding his breath. The parameters of acquiring the scan are as follows: slice thickness 1,25 mm, 120 kV, and 280 mA. The images were reconstructed on a dedicate CT work station (Advantage WorkStation 4.4, General Electric Medical System, Milwaukee, WI, USA with multi-planar-reconstruction (MPR), multiintensity projection (MIP), and volume rendering (VR) reconstruction (Figure 2). The images were evaluated by two radiological experts.

### 2.3. Management

**Figure 3:**
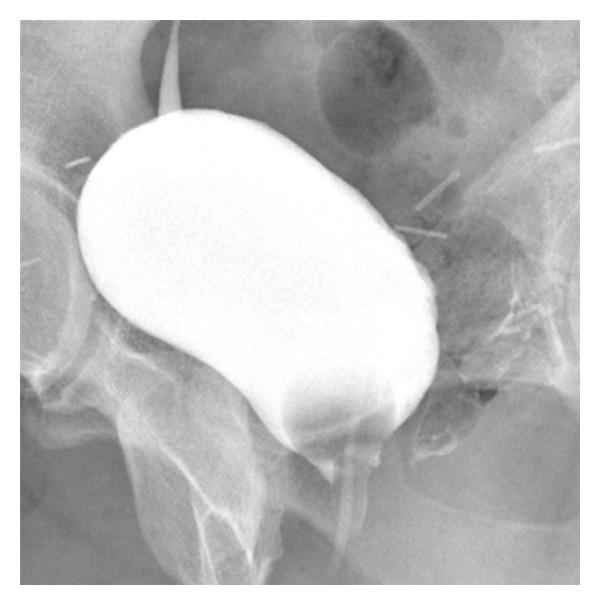
Retrograde cysto-uretrography after conservative treatment. Oblique radiograms of bladder after full repletion with contrast media, acquired 70 days later, depict the closure of the fistolous tract.

Since the CT scan confirmed that the fistula involved solely the urethra and not the bladder, it was possible to employ a conservative treatment by positioning a Foley catheter of monthly duration, in order to allow the urethra to rest. This treatment is also advisable for treating patients who cannot be operated on due to the presence of a cardiac comorbidity. During a followup 70 days later, the direct cystography with contrast media showed the closure of the fistulous tract (Figure 3). Still today, after 6 months, the patient is in good clinical condition and has not shown signs of a recurrence of the fistula. 

## 3. Discussion

The rectourethral fistula (RUF) is a possible but extremely rare complication of treatment of prostate cancer with “transrectal HIFU treatment.” 

HIFU is a technique that permits one to destroy the tumor located in the prostate by “bombarding” it with ultrasound at high intensity; this is aimed at precise points of the prostate thanks to an acoustic lens mounted on a transrectal and computer-driven ultrasound probe. The high energy in the target area brings about a noticeable increase of temperature (70–100°C), causing rapid coagulation necrosis of tissue within the target area without damaging the surrounding tissue [12, 13]. Two commercially available devices are currently in use for HIFU treatment of prostate cancer, Sonablate (Focus Surgery, Inc., Indianapolis, IN, USA), mostly trialled in Japan, and Ablatherm (EDAP SA, Lyon, France), widely used in Europe [14].

HIFU has been abundantly studied as a primary treatment (“primary HIFU”) for localised prostate cancer, in patients who are not suitable for radical surgery [13, 15–17]. Relatively recent studies performed on patients with local recurrent prostate cancer after primary radiotherapy (RT) have shown that HIFU is a valid alternative to salvage treatments currently in use (“salvage HIFU”). It is minimally invasive and results in fewer complications than other salvage techniques, such as radical prostatectomy, if used in well-chosen patients [12, 18]. The patients submitted to “salvage HIFU” should have a low or intermediate risk disease before radiotherapy. However, the ideal patient has not yet been clearly defined [12, 19]. Furthermore, it is necessary to find more sensitive and specific diagnostic exams, to detect local recurrence at an early curable stage, in order to carry out an efficacious salvage treatment with HIFU [12]. At any rate, although salvage HIFU seems conceptually promising, more detailed and longer followup is necessary to determine efficacy and survival benefit [12].

The RUF is a very rare complication, that can occur when HIFU is applied as the primary treatment for localised prostate cancer [20, 21]. Some studies show an occurrence of up to 2% but we must keep in mind that this percentage of fistulas was observed before the introduction of safety features such as rectal cooling and rectal safety margins with autodetection-driven alarms, or in patients with abnormal rectal anatomy [14]. Furthermore, technical improvements have allowed for a reduction in the HIFU time exposure (according to some studies, a 40% reduction) [16]. Due to technical improvements in the device and the use of transurethral resection (TURP) of the prostate before HIFU, the incidence of RUF seems to be considerably reduced [14, 15, 22]. Neoadjuvant androgen deprivation therapy may also be useful in larger prostates, especially because reduction of the target volume may increase the efficacy of the HIFU treatment [16].

Various studies demonstrate, instead, that the occurrence of fistulas following salvage HIFU after failed external beam radiotherapy (EBRT) is between 0% and 16% [12, 18, 19, 22–24]. Ahmed et al. affirm that the fistula could be a direct result of previous brachytherapy, EBRT, or both, and not directly related to the HIFU procedure. In fact, in a study conducted on 172 patients submitted to “primary HIFU,” no single RUF was found [21]. Meanwhile, an occurrence of 3–6% was noted in patients receiving salvage HIFU after failed EBRT. According to these authors, the high occurrence of RUF could be due to poor tissue viability and poor peri-prostatic blood supply subsequent to RT [25]. 

Marguet et al. and Chrouser et al. both reported that transrectal prostate biopsies might be another important factor in causing RUF; however, according to Ahmed et al., this is only true when combined with salvage treatment [25].

The fistulas seem to be more frequent in the case of repeated HIFU treatment [13, 16, 26]. Furthermore, they probably occur more often in patients with intestinal pathologies [13] or abnormal rectal anatomy [14].

The diagnosis of CVF using the common radiological techniques can be difficult. The ones that have given the best results have been barium enema (12.5–75%) [2–4, 8], cystography (44–90%), and cystoscopy (53.8%–69%) [2, 3, 8]. However, in the literature, the choice of the initial diagnostic study is controversial [1]. 

Plain abdominal radiography and intravenous urography (IVU) are of little diagnostic value because of their low sensitvity. Garcea et al. stated that the sensivity of plain abdominal radiography was 29% [27]. 

Cystography is a high sensitive diagnostic tool that provides diagnosis of CVF revealing the passage of contrast medium into the colon. In a retrospective study, cystography can diagnose CVF in 90% of patients [3], while in another retrospective study, cystography is the most sensitive study (44%) after cystoscopy (60%) [2]. 

Some believe that cystoscopy is the most effective diagnostic exam: it not only finds the fistula (30%) but also allows the identification of indirect signs (50%) such as bullous edema, localized inflammation, erythema, and granulation tissue; it also allows biopsy [6]. The first-level exams according to Daniels et al. are cystoscopy and urine cytology for faecal material. Subsequently, either barium enema should be used, for the preoperative investigation or CT, for patients with suspected extracolonic mass or malignancy [4]. 

Although double-contrast barium enema study is unreliable in demonstrating a fistula and reports a wide success range (12.5–72%), it is important for the determination of the underlying disease [1, 4]. It may reveal bowel diseases that cause enterovesical fistula, such as sigmoid colon diverticulitis or Crohn's disease [27]. 

Although a CT scan has higher sensitivity and specificity (86% and 91.3%, resp.), some authors believe that it often fails to demonstrate the fistulous tract [1]. Due to the technological improvements to the CT scan, others have lately shown that it is a highly sensitive technique (40–100%) and that, thanks to the 3D reconstructions, it identifies the fistulous tract (6–44%), gives precise anatomical and etiological information, and allows for the planning of possible surgeries with clear visuals of the surrounding structures. It also visualizes indirect signs such as gas, feces or oral contrast in the bladder, paravesical abcesses, and so forth [6]. Three-dimensional CT scans provide superior spatial detail [4].

Other diagnostic techniques have been suggested for the study of CVF, but the experience with them has been limited and the results have been poor. Magnetic resonance imaging, US [1], and 99 Tcm-DTPA that provides the flow rate across a fistula [4].Although direct radionuclide voiding cystography (DRVC) is generally used for detecting vesicoureteral reflux in pediatric patients [1] and it does not provide anatomic detail, it has revealed the presence of CVFs with dynamic imaging in a study on a patient. DRVC has demonstrated an extraurinary radionuclide activity in the sigmoid colon that was suggestive of CVF [1].

In our case, since the cystography is one of the diagnostic exams with greater sensitivity in confirming the suspicion of a fistula and the patient had already been catheterized, it was decided to carry out a retrograde cystourethrography as a first line radiological examination. This allowed us to document an “expansion of the contrast at the neck of the bladder” (Figure 1). 

When the bladder was full, through AP projection, a symmetrical expansion of the bladder was observed, with no alteration of the walls of the bladder, endoluminal protuberances, or diverticular formations. In this projection, it is possible to observe an expansion of contrast media in the left part of the urethra. Such an expansion could be better observed in the oblique projections, above all in those acquired after the space of a few minutes (Figure 1). In these, the fistulous tract was more visible and one could see a certain amount of contrast also in the rectum. It was not, however, possible to exclude with absolute certainty a partial involvement of the bladder. To confirm this finding, as well as to study the fistula's relation with the surrounding organs in order to carry out the appropriate treatment, a CT scan was acquired before emptying the patient's bladder. 

The CT is a sensitive diagnostic technique that is quick and noninvasive (more tolerated in patients than the barium/gastrografin enema [28]) which allows one to document the presence of the fistula [10]. In particular, CT with 3D reconstruction allows for a better and more complete image of the CVF's anatomical relations with the bladder and colon. It thus facilitates the planning of an eventual intervention [5, 10]. The signs most common in the presence of a fistula are air inside the bladder (90%), focal thickening of the bladder wall (90%), and/or of the adjacent intestinal wall (85%), extraluminal soft-tissue mass (75%), a passage of contrast media—administered orally or rectally—in the bladder (20%), and adherence of the intestinal wall to the bladder wall (25%) [28]. Though the CT allows one to evaluate minimal amounts of air—in contrast with the radiography—and can doubtless distinguish whether this is in the bladder or intestine, the presence of air in the bladder must at any rate be evaluated very carefully. This is due to the fact that it could be linked to bacterial infections or iatrogenic treatments, such as the positioning of a catheter [10]. Occasionally, the fistulization site can be identified or deduced by locating the focal thickening of the bladder and intestinal walls [28]. Otherwise, CT with oral contrast media (administered 2 hours prior to the procedure), or rectal contrast media, can be useful. i.v. contrast media must not be administered in such a way that the contrast media identified in the bladder would be attributed to the fistula [28]. The CT with oral contrast is advisable above all in evaluating CVF after bladder instrumentation [29]. In most of the studies, the CT is acquired with oral or rectal contrast media. The fistulous tract can be hard to identify through the CT with oral contrast media because of insufficient filling of the fistula [28]. In the initial state of a disease, the fistulous tract is usually thin and winding, and hard to identify even with the help of gravity, which favors the flow of the contrast in the bladder. However, in our case, the CT was acquired with contrast media in the bladder. It was thus possible to add to the information from the cystography—a highly sensitive technique for showing the fistulous tract—more information driven from the CT, without having to submit the patient to more contrast media. We thus obtained a better and more complete image of the anatomical relations that the CVF contracts with the bladder and colon, thus facilitating the planning of an adequate treatment [5, 10]. It is probable that the high quantity of contrast in the bladder—which, in contrast with the colon, is a closed sack—and the consequent increase in pressure in the bladder, in addition to gravity, favour a filling of the fistula by contrast media. This allows for the visualization of the fistula. Even if contrast media in the bladder can lead to a loss of information when studying the bladder and colon walls, we think it can be useful to carry out a pelvic CT scan with contrast in the bladder as a first exam or to complete a cystography. This exam, in fact, is useful above all in patients for whom it is necessary to obtain a precise study of the fistulous tract, unless they present suspected intestinal pathologies at the base of the fistula. 

## 4. Conclusion

In a patient undergoing cystography for rectourethral fistula, a CT scan—acquired with a full bladder, before emptying the bladder of means of contrast—can add more information without having to submit the patient to more contrast media. Even if contrast media in the bladder can lead to a loss of information when studying the bladder and colon walls, the pressure within the bladder and the force of gravity favors the filling of the fistula by contrast media facilitating its visualization and the planning of an adequate treatment.
